# Prevalence of arterial stiffness and the risk of myocardial diastolic dysfunction in women

**DOI:** 10.1042/BSR20160276

**Published:** 2016-10-27

**Authors:** Ute Seeland, Anna Brecht, Ahmad T. Nauman, Sabine Oertelt-Prigione, Mirjam Ruecke, Fabian Knebel, Verena Stangl, Vera Regitz-Zagrosek

**Affiliations:** * Institute of Gender in Medicine, Charité University Hospital, 10115 Berlin, Germany; † German Center for Cardiovascular Research (DZHK), 10115 Berlin, Germany; ‡ Department of Cardiology and Angiology, Charité University Hospital, 10117 Berlin, Germany; § Center of Cardiovascular Research (CCR), Charité University Hospital, 10115 Berlin, Germany

**Keywords:** arterial stiffness, augmentation index, diastolic dysfunction, pulse wave velocity, vascular dysfunction, vascular phenotypes

## Abstract

The present study determines the prevalence of vascular dysfunction and arterial stiffness (ASt) in a female urban population by measuring the brachial augmentation index (AIx) and aortic pulse wave velocity (PWV). The study tests the hypothesis that the measurement of AIx and PWV is useful in addition to that of traditional cardiovascular risk factors when assessing the risk for left ventricular diastolic dysfunction (LVDD). This cross-sectional study recruited 965 women aged 25–75 years from 12 districts of Berlin. The ASt indices, brachial AIx, aortic PWV and the central blood pressure were measured by an oscillometric method. A randomly selected subgroup (*n*=343) was examined by echocardiography. Trans-mitral inflow E/A ratio and diastolic mitral annulus velocity (*é*) were assessed. Questionnaires, medical history and blood sampling were used for the evaluation of individual risk factors. Normal vascular function was found in 55% of the women included. The prevalence of women with pathological AIx only (AIx ⩾ −10%, PWV normal) was 21.5%, whereas 17.9% were affected by increased AIx and PWV (AIx ⩾ −10%, PWV ⩾9.7 m/s), and 6% with only pathological PWV values. The prevalence of LVDD was 31.7%. LVDD was significantly associated with pathological PWV ⩾ 9.7 m/s [OR: 1.27, 95%CI: 1.02–1.57], age [OR: 4.17, 95%CI: 2.87–6.07] and a waist circumference >80 cm [OR: 3.61, 95%CI: 1.85–7.04] in multiple regression analysis. The high prevalence of markers for vascular dysfunction and ASt in a general female population and their importance as a mediator of diastolic dysfunction should encourage implementation of aortic PWV measurement to improve cardiovascular-risk assessment in particular to identify subclinical myocardial diastolic dysfunction.

## Abstract (precis)

The present study reports markers of vascular function among a general female population and shows that left ventricular diastolic dysfunction (LVDD) is significantly associated with pathological PWV (⩾9.7 m/s), a waist circumference >80 cm and age.

## INTRODUCTION

Detailed data about the vascular health of a general female population in Germany determined by the non-invasive measurement of vascular function due to the brachial augmentation index (AIx) and the arterial stiffness (ASt) parameter aortic pulse wave velocity (PWVao) are not available. The ‘Berlin Risk Evaluation in Women’ study was performed to determine the cardiovascular risk estimation and to measure the objective cardiovascular health of women living in Berlin [1]. Vascular health is influenced by many determinants, such as genetic predisposition and epigenetic modifications, age, cardiovascular risk factors and gender-specific socio-cultural circumstances. Protective conditions such as a favourable lifestyle have a positive effect on vascular function by powerful angioadaptive mechanisms.

The endothelium is a key regulator of vascular function [2]. Vascular dysfunction is the inability of the artery to dilate sufficiently in response to an appropriate endothelial stimulus and characterized by microvascular remodelling, decreased visco-elastic properties of the arteries and arterioles and abnormal total peripheral resistance (TPR) [3]. ASt of the larger arteries measured indirectly by the PWVao, is defined as the propagation of the flow wave and the reflection of the second pulse wave at the aortic bifurcation [4]. The AIx maintains the arterial tone and peripheral resistance, both are due to NO production and vasomotion of the medium and small arteries [5]. The AIx is defined as the pressure difference between the forward travelling pulse wave and the returning pressure wave in relation to the pulse pressure (PP), and is an indirect marker of the endothelial function. A faster propagation of the pressure wave and a faster return time of the second flow wave lead to the augmentation of the first pulse wave in systole [4]. These periodically repeating processes might be associated with elevated central aortic systolic blood pressure (SBPao) values. The Arteriograph device allows the measurement of SBPao at the same time as AIx and PWV [4]. The hypothesis, that the increase in left ventricular afterload might affect the left ventricular myocardial remodelling with impairment of diastolic function, is supported by the fact that elevated aortic PWV in older adults is associated with cardiovascular events such as cardiovascular mortality, coronary heart disease and stroke [6]. This issue is still a matter of debate [7]. Heart failure with preserved ejection fraction (HF-PEF) based on myocardial diastolic dysfunction is more prevalent in women than in men [8]. The importance of brachial AIx and aortic PWV as markers for vascular dysfunction and ASt and the association with asymptomatic organ damage, such as diastolic dysfunction, was not been studied previously in a female urban population and is the subject of the present study.

The 2013 ESC guidelines for the management of arterial hypertension call for more data from large studies in different populations to determine the potential role of the AIx and PWV measurements [9]. The first objective of the present study is to determine the prevalence of vascular dysfunction in a female urban population by measuring the brachial AIx and the aortic pulse wave velocity (PWV). The second objective is to test the hypothesis that the measurement of AIx and PWV is useful in addition to that of traditional cardiovascular risk factors when assessing the risk for asymptomatic organ damage, in particular to identify subclinical left ventricular diastolic dysfunction (LVDD).

## MATERIALS AND METHODS

### Study population

The Berlin Female Risk Evaluation (BEFRI) study is a community-based cross-sectional study. German-speaking women aged 25–75 years, represented in five equal age strata and from all 12 districts of the city of Berlin were included. We invited 3600 women to participate in the study. All women that expressed the intention to participate (*n*=1199) were sent the questionnaire, consent forms and further information materials by mail. Of these 1066 (88.9%) agreed to participate. For measurement of ASt indices *n*=965 and for echocardiography substudy *n*=473 were randomly selected proportional to the inhabitant density of each district. Each of the five 10-year age strata accounted for 20% of the total study sample. The exclusion criteria were physical or cognitive inability to participate in the study. More details are described in Oertelt-Prigione et al. [1]. The study was performed between September 2012 and November 2013. The study protocol was approved by the Ethics Committee of the Charité University Hospital Berlin (registration number: EA2/116/12) and all research conforms to the Declaration of Helsinki.

### Questionnaire

Participants were asked to answer a questionnaire to assess several behavioural aspects of their lifestyle and medical history. Detailed questions were asked about risk factors that play an important role for cardiovascular risk estimation in women, e.g. pregnancy complications, the onset of menopause and autoimmune diseases [10]. All patient data were pseudonymized.

### Physical examination

Clinical examinations were performed, and a standardized interview was conducted, focusing on the presence of acute and chronic cardiovascular symptoms. All women were weighed, and their waist circumference was measured in accordance with the World Health Organizations (WHO) standards. After a 10-min rest period, a 12-lead ECG was performed following a standardized procedure (AT-10 plus, Schiller).

### Measurement of arterial stiffness indices and haemodynamic parameters

Experimental conditions were arranged according to the international guidelines on how to conduct an investigation on arterial vascular stiffness [11]. All measures were performed in a supine position in a quiet, temperature-controlled room. Participants abstained from coffee and tobacco use on the day of the exam and relaxed for 10 min before initiation of the measurements. A non-invasive oscillometric method was used for measurement of ASt indices and haemodynamic parameters. The Arteriograph (TensioMed) has been validated invasively [4] and tested compared with tonometric and piezo-electronic techniques (e.g. SphygmoCor and Complior) [12,13]. The device allows simultaneous recordings of the ASt indices, the brachial AIx and the aortic PWV, as well as systolic and diastolic peripheral blood pressure (SBP, DBP), the central aortic blood pressure (SBPao) and aortic PP during cuff occlusion of the brachial artery. Pulsatile pressure changes in the brachial artery are detected by plethysmography, and pressure pulse configurations are transmitted to computer software that analyses ASt indices. The brachial AIx is calculated from the difference in amplitude (pressure difference) between the first and reflected second wave in relation to the PP, based on the formula: AIx %=[(*P*2 − *P*1)/PP] × 100. The brachial AIx levels are defined as normal (<−10%), high (−10% to +10%) and pathological (>+10%) for all age strata [14,15]. Aortic AIx correlates with brachial AIx using the regression equation *y*=0.506*x* + 37.636 [4]. The AIx is regarded as an indirect marker of the endothelial function which maintains the arterial tone and peripheral resistance [5]. The aortic PWV (m/s), the time difference between the start of the first pulse wave and the beginning of the reflected wave, is calculated based on the formula: jugulum–symphysis distance/(return time/2) [m/s]. The distance travelled by the waves is assimilated as the distance between the jugulum and the symphysis and measured individually with a tape. Aortic PWV levels are characterized as normal (<9.7 m/s), high (9.7–9.9 m/s) or pathological (>10 m/s) for all age strata, supported by outcome data [14,16]. For data quality control, the standard deviation (S.D.) was calculated from every heartbeat during a period of 8 s, and the inclusion criterion was defined as a PWV with a S.D. < 1.3 m/s. Three consecutive measurements were performed and the values with the lowest S.D. (highest technical quality) were used. The algorithm for estimating SBPao is based on the physiological relationship between DBP, mean arterial pressure (MAP) and peripheral and central AIx [4]. For measurement of the ankle-brachial index (ABI), blood pressure was taken from the right and left lower leg in addition to the upper arms. A value ≤0.9 indicates peripheral artery disease (PAD) [17].

We categorized all women into four vascular phenotype groups, according to brachial AIx and aortic PWV values:

**Table:** 

	AIx normal	AIx high/pathological
	(<−10%)	(⩾−10%)
**PWV normal** (<9.7 m/s)	(a) Normal vascular function (healthy)	(b) Vascular dysfunction
**PWV high/pathological** (⩾9.7 m/s)	(c) Arterial stiffness	(d) Vascular dysfunction and arterial stiffness

### Laboratory data

Laboratory measurements were made on non-fasting blood samples and processed within 24 h of collection. Full blood counts, GFR (glomerular filtration rate), lipid metabolism, HbA1c (glycated haemoglobin), BNP (B-type natriuretic peptide) and sexual hormones were quantified by an external laboratory (Hospital Laborverbund Berlin-Brandenburg, Bernau, Germany).

### Standard definitions for risk factor assignment

Patients were classified according to their risk factor profile, as determined by reported hypertension and their diabetes status. For the definition of dyslipidaemia, the ratio of total cholesterol (CHOL)/high-density lipoprotein cholesterol (HDL-C) was used, with a cut-off value >5. Overweight was defined as BMI ⩾ 25 kg/m^2^. Waist circumference was defined as abnormal if >80 cm. Normal ranges are ≤100 pg/ml for BNP and 4–6% for HbA1c. Smoking status was coded as current, past or never. Rheumatologic diseases, pregnancy complications and the age of onset of menopause were asked in detail. Menopause was defined as the absence of regular bleeding with consideration for circumstances that could have an effect on the bleeding pattern. Parity was defined as having any number of children. Pregnancy complications included circulatory disorders (such as pregnancy hypertension), pre-eclampsia, eclampsia, HELLP (haemolysis, elevated liver enzyme levels, low platelet), premature birth, intrauterine growth retardation, intrauterine fetal death and placental insufficiency. Furthermore, drug names were recorded and assigned to categories.

### Echocardiography

Echocardiographic parameters were measured for studying potential asymptomatic left ventricular organ damage. Standard transthoracic echocardiography (TTE) was performed with a GE Vivid E9 system (GE Healthcare) and an M5S probe. Data were acquired with the subjects at rest. Examinations were performed by experienced cardiologists according to Lang et al. [18]. Standard parameters of end-systolic and end-diastolic dimensions and left ventricular ejection fraction (LVEF) were measured. Pulse-wave (PW) Doppler of trans-mitral inflow was performed in the apical 4-chamber view. Mitral inflow includes peak early filling (E-wave) and late diastolic filling (A-wave) velocities, E/A-ratio, deceleration time (DT) of early filling velocity and left ventricular (LV) isovolumic relaxation time (IVRT). The average septal and lateral diastolic mitral annulus velocity (*é*) was assessed by tissue Doppler imaging (TDI) of the basal segments [19]. Diastolic function was graded according to the recommendations of the American Society of Echocardiography (ASE) [20]:

• DD0=normal diastolic function: E/A ⩾ 1, average *é* > 9 cm/s;
• DD1=impaired relaxation or mild DD: E/A < 1, average *é* ≤ 9 cm/s;
• DD2=pseudonormal function or moderate DD: E/A ⩾ 1, average *é* ≤ 9 cm/s.

Classification of all patients into the above three grades of diastolic function was performed independently by two echocardiography experts based on all available data.

### Statistical analysis

Descriptive statistics comprised medians with 25% and 75% quantiles (IQR=interquartile range) or means and S.D. where appropriate for continuous data and proportions for categorical data. Differences in categorical and continuous data were determined using Chi-square (*χ*^2^), *t* test or ANOVA. Age-adjusted *p*-values for comparing groups of vascular function/dysfunction were calculated using multinomial regression models. Pearson's correlation coefficients were used to test the correlation between continuous, sufficiently normally distributed (mono-modal, |skewness|<1) variables. Variables showing significant association with the outcome variable ‘diastolic dysfunction’ were further analysed in multiple logistic regression models with pathological characteristics as dependent variables. Results from the multiple logistic regression analyses are presented as odds ratios (ORs) with 95% confidence intervals (CI) and adjusted *R*^2^. A two-sided significance level of α=0.05 was used. Data analyses and statistical calculations were performed with SPSS 20 software.

## RESULTS

One thousand sixty-six women participated in the ‘Berlin Risk Evaluation in Women’ study and signed consent forms. Measurements of ASt indices and haemodynamic data were made on 965 women. Data from 160 women had to be excluded because of either an insufficient S.D. of PWV (⩾1.3 m/s) of sequential pulse wave velocity records or atrial fibrillation. Therefore, 805 (83%) records complied with the requirements of data quality control. The 343 participants included in the subgroup analysis for the measurement of LVDD were representative of the total study cohort with regard to age strata (Supplementary flow chart).

The total cohort was categorized according to the AIx brachial and aortic PWV measurements. Women with normal vascular function were separated from those with vascular dysfunction. High/pathological brachial AIx values were measured in 317 women from the available sample of 805 (39.4%), and high/pathological aortic PWV values were found in 191 (23.7%). Although 54.8% had normal vascular function (*n*=441, group a), the prevalence of vascular dysfunction was 21.5% (*n*=173, group b). Moreover, 17.9% of the study population were affected by ASt (*n*=144, group c), and 47 women (5.8%, group d) had pathological values of PWV but normal AIx (Table 1). High/pathological AIx and PWV values were more frequently measured in postmenopausal women. Numbers are shown in detail for each age stratum in Table 2. Mean (S.D.) age at onset of menopause was calculated to have been 49 (±7) years.

**Table 1:** Prevalence of women with normal vascular function and dysfunction Group a: normal vascular function, group b: vascular dysfunction, group c: vascular dysfunction (VD) + arterial stiffness and group d: arterial stiffness. Absolute numbers and percentage of total (*n*=805) are shown.

	AIx normal	AIx high/pathological *n* (⩾−10%)	
**PWVao normal**	(a) 441 (54.8%)	(b) 173 (21.5%)	614 (76.3%)
**PWVao high/pathological** (⩾9.7 m/s)	(d) 47 (5.8%)	(c) 144 (17.9%)	191 (23.7%)
	488 (60.6%)	317 (39.4%)	805 (100%)

**Table 2:** Distribution of normal, high and pathological values of the brachial AIx and aortic PWV Numbers are given as absolute values and percentage of each age stratum. High brachial AIx is defined as −10% to +10% and pathological as >+10%. High aortic PWV is defined as 9.7–9.9 m/s and pathological as >10 m/s.


AIx/PWVao\Age strata (years)					
	25–34	35–44	45–54	55–64	65–75
	*n*=185	*n*=176	*n*=158	*n*=137	*n*=149
**AIx brachial**					
Normal *n* (%)	176 (95.1)	145 (82.4)	82 (51.9)	47 (34.3)	38 (25.5)
High *n* (%)	6 (3.2)	24 (13.6)	31 (19.6)	40 (29.2)	41 (27.5)
Pathological *n* (%)	3 (1.6)	7 (4.0)	45 (28.5)	50 (36.5)	70 (47.0)
**PWVao**					
Normal *n* (%)	181 (97.8)	164 (93.2)	122 (77.2)	76 (55.5)	71 (47.7)
High *n* (%)	0	5 (2.8)	6 (3.8)	10 (7.3)	10 (6.7)
Pathological *n* (%)	4 (2.2)	7 (4.0)	30 (19.0)	51 (37.2)	68 (45.6)

Characteristics about age, menopausal status, medical history, traditional and more women specific cardiovascular risk factors, for the four vascular phenotype groups are presented in Table 3. Age adjusted comparison of data between all groups show significant differences in the postmenopausal status, the percentage of women with BMI ⩾ 25 kg/m^2^ and those with waist circumference >80 cm.

**Table 3:** Cardiovascular risk factors and medical history data by group Numbers are given as absolute values and percentage unless otherwise stated (*n* = 805). Unadjusted comparisons between all groups were calculated with ANOVA, *t* test or Chi-square-tests. a*p*-value adjusted for age using multinomial regression. MI, myocardial infarction; CAD, coronary artery disease; S.D., standard deviation.

	(a) Normal vascular function	(b) Vascular dysfunction	(c) VD + arterial stiffness	(d) Arterial stiffness	*p* (unadjusted for age)	a*p* (adjusted for age)
***n***	441	173	144	47		
**Age** mean (S.D.)	41 (12)	56 (12)	62 (9)	56 (12)	<0.001	–
**Postmenopausal** (⩾49 years), *n* (%)	92 (20.9%)	123 (71.1%)	132 (91.7%)	33 (70.2%)	<0.001	0.035
**Cardiovascular risk factors**						
Hypertension, *n* (%)	46 (10.5%)	52 (30.1%)	62 (43.1%)	15 (32.6%)	<0.001	0.616
Diabetes mellitus, *n* (%)	11 (2.5%)	8 (4.6%)	10 (6.9%)	6 (12.8%)	0.004	0.190
CHOl/HDL ratio >5, *n* (%)	40 (9.1%)	20 (11.6%)	23 (16.0%)	5 (10.6%)	0.147	–
Current smoking, *n* (%)	145 (33.0%)	37 (21.5%)	30 (20.8%)	10 (22.2%)	0.004	0.568
Overweight (BMI > 25), *n* (%)	120 (27.2%)	53 (30.6%)	70 (48.6%)	32 (68.1%)	<0.001	<0.001
Visceral fat (waist >80 cm), *n* (%)	127 (28.8%)	83 (48.0%)	90 (62.5%)	34 (72.3%)	<0.001	0.011
**Medical history**						
Diseases: CAD or any other: MI, PAD, heart failure, stroke, TIA	11 (2.5%)	7 (4.0%)	11 (7.6%)	4 (8.5%)	0.016	0.240
Parity, *n* (%)	243 (55.1%)	129 (74.6%)	120 (83.3%)	33 (70.2%)	<0.001	0.489
Pregnancy complications, *n* (%)	38 (8.6%)	25 (14.5%)	27 (18.8%)	7 (14.9%)	0.006	0.330
Rheumatologic diseases, *n* (%)	41 (9.3%)	28 (16.2%)	27 (18.8%)	12 (25.5%)	<0.001	0.458

Haemodynamic parameters were measured simultaneously with the vascular function indices. Results are shown in Table 4 with significant differences in age adjusted values between all groups for SBPao, mean-, systolic- and diastolic arterial pressure and heart rate.

**Table 4:** Haemodynamic data by group Numbers are given as mean values and standard deviation (S.D.) for *n*=805. Sys, systolic blood pressure; Dia, diastolic blood pressure; HR, heart rate; *p*=unadjusted differences between all groups were analysed using ANOVA; a*p*=adjusted for age using multinomial regression.

	(a) Normal vascular function	(b) Vascular dysfunction	(c) VD + arterial stiffness	(d) Arterial stiffness	*p* (unadjusted for age)	a*p* (adjusted for age)
**SBPao** (mmHg)	109 (13)	136 (20)	143 (21)	125 (16)	<0.001	<0.001
**MAP** (mmHg)	86 (11)	96 (14)	100 (14)	95 (10)	<0.001	<0.001
**Sys** (mmHg)	118 (13)	131 (19)	137 (20)	131 (15)	<0.001	<0.001
**Dia** (mmHg)	70 (10)	79 (13)	81 (12)	77 (9)	<0.001	<0.001
**PP** (mmHg)	48 (8)	52 (11)	56 (12)	53 (11)	<0.001	0.094
**HR** (min^−1^)	68 (10)	65 (8)	67 (8)	79 (10)	<0.001	<0.001

### Myocardial diastolic dysfunction and arterial stiffness indices

Echocardiography was performed in a representative subgroup of 343 randomly selected women to determine LV function, in particular diastolic function, which is related to myocardial relaxation, passive LV properties and the myocardial tone. The prevalence of diastolic dysfunction (DD1 + DD2) was 31.7% (*n*=109) and 68.3% (*n*=235) had normal diastolic function (DD0). Using multiple logistic regression analysis for age adjustment of the data, results show significantly higher central blood pressure values (SBPao [mmHg] DD1 + DD2: 131.8 ± 19.5, DD0: 114.4 ± 19.1; *P*=0.036) and increased aortic PWV ([m/s] DD1 + DD2: 9.9 ± 1.9, DD0 8.0 ± 1.6; *P*=0.034) in women with LVDD compared to women with normal myocardial function. The brachial AIx values were measured in the high range (mean −3.3%) in the group with diastolic dysfunction and in normal range (mean −26.7%) in the healthy group. However, age adjusted *p*-value was not significant between the groups for brachial AIx (Table 5).

**Table 5:** Arterial stiffness indices and central blood pressure of women with normal diastolic function (DD0) and mild diastolic dysfunction: impaired relaxation (DD1) and pseudonormal function (DD2) DD0: E/A ⩾ 1, average *é* > 9 cm/s; DD1: E/A < 1, average *é* ≤ 9 cm/s; D2: E/A ⩾1, *é* ≤ 9 cm/s. DD1 and DD2 are calculated in one group because of less numbers in DD2 (*n*=16). *é*=average of septal and lateral diastolic mitral annulus velocity (TDI). Mean values and S.D.; unadjusted *p*-values derived by using *t* test for independent samples, a*p*-value adjusted for age using multiple logistic regression. Total *n*=343.

	Normal diastolic function (DD0)	Diastolic dysfunction (DD1 + DD2)	*p* (unadjusted)	a*p* (adjusted for age)
	235 (68.3%)	109 (31.7%)		
**SBPao** (mmHg)	114.4 (19.1)	131.8 (19.5)	<0.001	0.036
**AIx brachial** (%)	−26.7 (27.8)	−3.3 (23.6)	<0.001	0.127
**PWVao** (m/s)	8.0 (1.6)	9.9 (1.9)	<0.001	0.034
**PP** (mmHg)	48.1 (7.8)	53.4 (10.2)	<0.001	0.177

To answer the question of the usefulness for the measurement of brachial AIx and aortic PWV in addition to the traditional cardiovascular risk factors with regard to the risk for LVDD, brachial AIx and aortic PWV have been added to the multiple logistic regression model, with DD0 and DD1 + DD2 as the dependent variables. Known classical cardiovascular risk factors and other more women-specific risk factors with significant coefficients in univariate analysis were added to the model. Table 6 (model 2) shows the association of age (OR: 4.17, 95%CI: 2.87–6.07), waist circumference >80 cm (OR: 3.61, 95%CI: 1.85–7.04) and high/pathological aortic PWV levels (OR: 1.27, 95%CI: 1.02–1.57) with LVDD.

**Table 6:** Multiple logistic regression analysis for diastolic dysfunction Categorical variables: hypertension, visceral fat, parity, pregnancy complications, family history of hypertension. Continuous variables: AIx brachial, PWVao, *Age: age in decades=age in years/10. AIx brachial and PWVao were forced into the model.

	Model 1 (full model) OR (95%CI)	Model 2 (only significant variables and AIx brachial and PWVao) OR (95%CI)
	*n*=343, *R*^2^=0.65	*n*=343, *R*^2^=0.63
**AIx brachial**	0.99 (0.97–1.00)	0.99 (0.97–1.00); *P*=0.123
**PWVao**	1.22 (0.97–1.52)	1.27 (1.02–1.57); *P*=0.033
**Hypertension**	1.79 (0.74–4.36)	–
**Visceral fat** (waist > 80 cm)	2.82 (1.20–6.65)	3.61 (1.85–7.04); *P*<0.001
**Overweight** (BMI > 25 kg/m^2^)	1.36 (0.55–3.34)	–
**Parity** (any no. of children)	1.97 (0.87–4.44)	–
**Pregnancy complications**	0.37 (0.12–1.11)	–
**Family history hypertension**	1.03 (0.48–2.18)	–
Age*	4.19 (2.79–6.30)	4.17 (2.87–6.07); *P*<0.001

## DISCUSSION

The first stage of the cardiovascular aging continuum is the loss of arterial vascular elasticity. Cardiovascular risk factors exacerbate this process and promote the development of atherosclerosis and the premature aging of the arteries [21]. Vascular dysfunction and increased ASt are common and relevant problems of the female general population as shown by the high prevalence of pathological AIx with 21.5% and abnormal measurements of PWV with 17.9% in the present study. In order to assess whether the measurements of brachial AIx and/or aortic PWV are associated with early asymptomatic organ damage, we measured LV diastolic function in all vascular phenotype groups: women with normal vascular function, women with vascular dysfunction due to increased peripheral vascular resistance and endothelial dysfunction and women with ASt. The prevalence of LVDD in the randomly selected study subcohort was 31.7%. None of the women had signs of congestive heart failure. In addition to the known cardiovascular risk factor age, we identified that high/pathological PWV ⩾9.7 m/s and waist circumference >80 cm, a surrogate parameter for expanded visceral fat, are associated with LVDD. An echocardiographic examination for the diagnosis of diastolic dysfunction is not performed in the clinical routine. Therefore, an easy-to-measure parameter such as the PWV is useful in otherwise asymptomatic patients to provide the indication for this diagnostic approach. Identifying these women with diastolic dysfunction in an early clinical stage is important, aiming to prevent heart failure development with preserved ejection fraction (HF-PEF) [22,23].

Albu et al. [24] provided evidence that ASt as measured by aortic PWV is a risk factor for LVDD in postmenopausal women, independent of other traditional risk factors. Our study data support those findings. Yet we examined a larger cohort including premenopausal and postmenopausal women, and we expanded upon the number of risk factors. In addition to the classic cardiovascular risk factors, the medical history of women-specific risk factors with significant coefficients in univariate analysis–such as pregnancy complications, parity and rheumatologic diseases–were added to the regression analysis models. Moreover, diastolic function was graded according to the recommendations of the American Society of Echocardiography (ASE) including average *é*, which is highly correlated with LVDD than the E/A ratio alone.

Recently the EPOGH investigators [25] determined Doppler indexes reflecting LV systolic and diastolic dysfunction and ASt in 1233 randomly recruited study participants (51.7% women). In line with the data of our study central PP, augmentation pressure and PWV, were on average significantly higher in the diastolic dysfunction group with elevated filling pressure when compared to participants with normal diastolic function or with impaired relaxation. However, the authors did not perform a gender-specific analysis of their data. Supported by the findings of Libhaber et al. [26] data should be analysed by sex. They demonstrated an association of PWV with LV mass index and LV wall thickness independent of blood pressure and additional confounders in a never treated population sample of women, but not men. Sex differences of arterial characteristics and vascular function should be considered in further studies with the aim to study sex-specific CV-risk factors affecting AIx and PWV.

Tarnoki et al. [27] provided a further important contribution regarding heritability of ASt. The authors measured central SBP and ASt in 154 monozygotic and 42 dizygotic twin pairs with Arteriograph device. They observed that age-, sex- and country-adjusted heritability was 60% for central SBP, 50.1% for aortic PWV and 46.8% for brachial AIx. The study data indicate that the heritability of central SBP and the ASt indices is moderate. Unshared environmental effects account for large portion of the variance in this and in our study, especially for the brachial AIx values. Further studies should focus to the role of epigenetic modulation. These findings highlight the genetic and environmental aetiology of vascular aging and the importance of early atherosclerosis screening, detection and prevention in high-risk patients. These findings highlight the genetic and environmental aetiology of vascular aging and the importance of early atherosclerosis screening, detection and prevention in high-risk patients.

The present study contains some limitations. The role of age regarding the definition of normal- or reference values for describing the normal elasticity and impedance of the arteries, needs careful consideration. ASt increases with age and BP; as these are the major determinants of PWV. However, PWV values are not simply associated with the increase in BP by age but include additional information about the function of the aortic wall [28]. ‘Vascular’ aging is an individual and continuing process as described by O'Rourke et al. [21]. Normal values are known for younger people with a mean age of 33 years without any CV-risk factors. The approach to define the exact values representing normal and abnormal PWV values by each year of age is limited as long as outcome data of longitudinal studies are missing. Reference values are associated with a higher risk of early organ damage and used in the present study. We choose a three-step scale with ‘normal’, ‘high’ and ‘pathological’ for AIx and PWV. The limit values for pathological PWV used in the present study correspond to these reference values of the Arterial Stiffness Collaboration established in a European population for subjects 50–69 years of age with grade 1 hypertension (<160/100 mmHg) and for >70 years of age with high normal blood pressure (<140/90 mmHg) [28]. With this approach, the prevalence of women with ASt would might be underestimated in the younger age strata. Concerning the interpretation of the study data, we are aware that we cannot determine causality but associations and probabilities with the cross-sectional study design.

The study data provide evidence for the association of PWV ⩾ 9.7 m/s and diastolic dysfunction. This is in line with the general expert opinion that PWV ⩾ 10 m/s is associated with a high risk for early organ damage [9].

The question that has not yet been conclusively answered is the potential role of the brachial AIx as a risk factor for cardiovascular events. The brachial AIx is a parameter for composite vascular function, including not only static properties such as distensibility but also dynamic ones such as endothelial function and TPR [29]. In the present study, pathological AIx was not associated with LVDD. Assuming that pathologic AIx usually precedes pathological PWV, LVDD would not be the best outcome variable to answer the question of the impact of AIx on early organ damage. Another explanation could be that the parameters AIx and PWV are more independent of each other due to different risk factors affecting brachial AIx or aortic PWV values. Detailed risk factor analysis would help to answer this question and will be considered in this cohort.

Furthermore, the study data stressed the fact that measurement of waist circumference >80 cm is associated with impaired left ventricular diastolic function in addition to age and increased PWV. This finding is consistent with recently data published by Sekiguchi et al. [30]. They showed that higher waist circumference and age are associated with abnormal LV myocardial relaxation in normal-weight subjects. The age-adjusted data given in our study, confirm the association of statistically significantly higher central blood pressure (SBPao) values of women with diastolic dysfunction compared to those with normal myocardial function. Recently, Herbert et al. [31] published reference values for central blood pressure (SBPao) and its amplification in a general healthy population according to age, sex and peripheral brachial blood pressure, with a wide geographical representation.

In summary these data support the opinion that the consideration of these easy to measure parameters such as the age, waist circumference measurement and the PWV, should lead to a recommendation on the echocardiographic diagnostic to assess women with diastolic dysfunction before developing symptomatic HF-PEF.

## CONCLUSION

The study data show that pathological aortic PWV values ⩾ 9.7 m/s are associated with LV diastolic dysfunction, in addition to waist circumference >80 cm and the age. The high prevalence of vascular dysfunction and ASt in a general female population and the importance of arterial function as a mediator of myocardial diastolic function support the usefulness of assessment of aortic stiffness as a marker of cardiovascular disease.

## Abbreviations

AIx: augmentation index
ASt: arterial stiffness
BNP: B-type natriuretic peptide
HDL-C: high-density lipoprotein cholesterol
HF-PEF: heart failure with preserved ejection fraction
LVDD: left ventricular diastolic dysfunction
MAP: mean arterial pressure
OR: odds ratio
PAD: peripheral artery disease
PP: pulse pressure
PWV: pulse wave velocity
SBPao: central aortic systolic blood pressure
TPR: total peripheral resistance
